# The Microbe of Pleuro-pneumonia

**Published:** 1898-08

**Authors:** 


### SELECTIONS.

### THE MICROBE OF PLEURO-PNEUMONIA.

By Messieurs Nocard and Roux.

The essential lesion of contagious pleuro-pneumonia of bovine animals consists in the distention of the meshes of the interlobular conjunctive tissue by a great quantity of albuminous, yellowish, and limpid serum, which is very virulent. Suppose we inoculate a drop of it into the subcutaneous cellular tissue of a healthy cow. After an incubation, which is never less than eight days, but which may go on as long as twenty-five days and more, we shall see appearing an inflammatory, hot, hard, and painful lump, whose dimensions will vary a great deal according to the seat of inoculation, and also according to the subjects inoculated. If the virulent serum is inoculated under the skin of the trunk, of the head and shoulders, or of the upper part of the limbs, it causes a considerable swelling, which spreads rapidly and is often followed by death; this is accompanied by an intense fever. On autopsy the meshes of the cellular tissue are found distended by an enormous quantity of yellow limpid serum, here and there coagulated into gelatinous and quivering masses. The exudation is sometimes so plentiful that several litres of virulent serum may be collected; but widespread though it is, it never attacks the conjunctive compartments of the lung; a little serous exudation may be found in the pleural cavity, but visceral lesions are never observed; death is, then, the result of an intoxication. Certain subjects resist. After a few days the swelling, which has so far been hot, hard, and painful, remains stationary, then it sinks and gradually disappears without leaving any trace. These subjects will ever afterward be refractory to the effects of virulent inoculation as to those of natural contagion. This happy outcome is the rule when inoculation takes place far from the trunk, at the extremity of the tail. For example, where the density of the tissues and the low local temperature do not favor active spreading of the virus. The swelling consequent on the inoculation is always analogous to that described above; but it remains limited in extent, and disappears gradually, leaving the animal refractory to either natural or experimental contagion. Sometimes, however, the exudation is so abundant and exercises on the tegument such a tension that it brings on mortification and the falling off of a more or less long piece of tail. Sometimes, finally, but rarely (1 or 2 per cent.), the swelling instead of remaining limited to the extremity of the tail, rapidly rises up along the organ and attacks the cellular tissue of the rump and pelvis; death generally follows, and the regions attacked are, on autopsy, shown to be infiltrated with a considerable quantity of serum similar to that of the lung in the natural disease.

The pleuro-pneumonic serum, so virulent for animals of the bovine species, is without action on those of the other species—the goat, sheep, dog, pig, rabbit, guinea-pig, and barn-door fowls, without any harm stand the subcutaneous or intraperitoneal injection of large doses of virulent serum. These facts were established by Willems as early as 1850; he deduced from them the rules of an efficacious prophylaxy. But Willem's method of inoculation, which has rendered great service, is not without inconveniences. It necessitates the deposit of a drop of pulmonary serum in the cellular tissue of the caudal extremity of the animal to be immunized. Now, this pulmonary serum becomes very easily spoiled; it rapidly becomes putrid, and the putrefaction destroys its virulence. One must, therefore, have a fresh lung for inoculation. Generally, a pleuro-pneumonic animal is slaughtered at the moment of the operation; but sometimes the animal slaughtered has nothing but an old lesion, in which the serous exudation may have lost its virulence, when it is not entirely wanting; sometimes also, especially when it is a question of making truly preventive inoculations, there is no pleuro-pneumonic cow to slaughter. A real step in advance was taken on the day when M. Pasteur taught us to collect in its purity the serum which distends the conjunctive compartments of the pleuro-pneumonic lung, and, above all, to produce great quantities of virulent serum by inoculating into the calf a drop of pulmonary serum. From that time forward it was possible to make stores of virus and send it abroad as required. Yet the problem has not been entirely solved; the pleuro-pneumonic serum, even when collected in a pure state, loses its virulence fast enough; after a month, or six weeks at most, the inoculation ordinarily remains without effect. In order to be sure of being able to satisfy all wants, centres for the production of virus must be established in which, each month at least, new subjects are inoculated. That is what has been accomplished at great expense in one or two countries. The determination of the specific agent of the pleuro-pneumonic virus, its isolation, and its culture would therefore, constitute a considerable step. Unfortunately, all who have devoted themselves to this study—and their number is legion—have failed. We had on our part made numerous attempts and for a very long time. They all remained fruitless of result. When it has been collected in a pure state in the peri-lobular or subpleural lymphatic sacs, the most virulent pleuro-pneumonic serum may be sown in all the usual media in the air or in a vacuum, without giving any culture. Likewise it was found impossible to color in it by the usual processes any microbian element. We had, then, given up every attempt at cultivation when the *Memoire* of MM. Metchnikoff, Roux, and Salimbeni on the toxin and antitoxin of cholera was published. The results obtained by them with the help of collodion restored our hopes of success. We will in a few words recall the principles and technique of this ingenious method of cultivation.

Small sacs of collodion of a very thin texture are prepared; after they have been sterilized on the autoclae a few cubic centimetres of bouillon are introduced into them which has been sown previously with a dash of virulent liquid; they are closed up tight, then they are introduced into the peritoneum of a fresh and healthy animal—a guinea-pig, dog, sheep, cow, etc. One soon learns how to perform all these operations simply, and the animal never seems to suffer for an instant either from the operation or from the presence of the sacs in the peritoneal cavity. After a time, varying from some days to several months, according to the nature of the microbe studied, the animal is slaughtered; the sac is found lodged in some corner of the peritoneal cavity enveloped by a more or less thick layer of fibrin and cellules, or of young fibrous tissue, from which it may be easily enucleated. When the subject and the liquid of the cultivation have been suitably chosen, surprising results are obtained, the interpretation of which is, however, easy. The coating of collodion presents an insurmountable barrier to the microbes as well as to the cellules. The microbes cannot leave the sac, but they may multiply in it in all security, for the cellules, not being able to get inside the microbes, are protected from the phagocytosis. On the other hand, this coating, which is inaccessible to the microbes and to the cellules, can be permeated by liquids as well as by dissolved substances; it forms a perfect osmotic membrane; where it lies changes take place which have a profound effect in modifying the primitive composition of the liquid imprisoned. Substances elaborated by the microbe may leak through to the outside, and when they are sufficiently sensitive they may cause the death of the subject, or more or less grave accidents in the way of poisoning without a single microbe having attacked the tissues; in any case it is a condition which is favorable for the cultivation, the microbic auto-poisoning being diminished, if not suppressed; finally, products which have come from the organism of the subject penetrate into the sac products, which may be favorable to the cultivation of the microbe. That is the commonest case. Also, when the sac is opened there is found in it a cultivation of extraordinary richness. "This method of procedure," say the authors, "is very convenient for preserving the fragile microbes, and it succeeds with many kinds." Pehaps, then, it would succeed with the pleuo-pneumonic virus. The result confirmed our expectations.

Sacs of collodion filled with a bouillon which had been previously sown with a little pleuro-pneumonic serum were carefully closed up and inserted into the peritoneal cavity of rabbits. After fifteen to twenty days these sacs contained an opaline liquid, a little hazy and slightly albuminous. This liquid contained none of the cellules or bacteria cultivable in the usual bouillons. On the other hand, microscopic examination showed in it, at a very high magnifying point (about 2000 diameters) and in a very powerful light, an infinite number of refringent and mobile points so attenuated that it was impossible, even after coloring, to determine their shape exactly. If anyone chose to insert into the peritoneum of the same rabbit a second sac of collodion enclosing some bouillon, identical but not sown, he might assure himself that the modifications undergone by the liquid of the first sac were not due purely and simply to the osmotic changes which took place at the coating; he would, in fact, find that the liquid of the test sac had preserved its original transparency and limpidity. In reality the mobile and refringent points in the liquid sown, so numerous that in spite of their extreme firmness they rendered the liquid opalescent, are living beings which have gone on swarming to infinity, favored by modifications undergone by the liquid cultivation and thanks to the obstacles hrown in the way of phagocytic action by the coating of collodion. What proves this is that if there are inserted into the peritoneum of a second rabbit two sacs of collodion which have been sown, the first with a dash of the opaline liquid thus obtained, the second with several drops of the same liquid, which have been previously heated, the latter acts exactly the same as the test-sac just mentioned; its contents remain limpid and transparent, whilst the other soon presents opalescence and the numerous refringent points described above; the heating had killed the germs sown. With the opaline liquid given by the fertile sac of the second rabbit, and thus successively identical results continue to be obtained. But it is prudent to make several sacs for each transfer, rupture of the sacs taking place frequently. Generally, the rabbits have become very thin when they are slaughtered; sometimes even they succumb before the day fixed for the autopsy; they are then in a state of profound cachexia; they are nothing but skin and bones. Autopsy, however, reveals no appreciable organic lesion; the blood and pulp of the parenchymas when sown in various media, even in sacs of collodion, gave no cultivation; it is, therefore a question in all probability of a poisoning due to the diffusion outside the sac of products elaborated by the microbe; they cannot in all cases be attributed to digestive troubles (or others) which the presence of the sac might have produced, being a foreign body. When the bouillon put in the sacs has not been sown the rabbits may receive several sacs and keep them for several months without showing the least signs of uneasiness, without losing a gramme in weight.

It seemed to us, beside, that these accidents were more marked and the cachexia more serious in proportion, as the sacs introduced after sewing were more numerous, their capacity was greater, and the cultivation arrived at was richer. Here, then, is a fresh example of an animal which is very sensitive to the toxins of a microbe, against which, as a microbe, it is yet quite refractory. We tried several times to obtain cultures in sacs in the guinea-pig, but never succeeded in doing so; even after remaining for six weeks in the peritoneum of the guinea-pig the most plentifully sown liquid was found as limpid as at the beginning. We have, therefore, to do with a special microbe which has multiplied in successive cultivations in the particular medium which the osmotic changes have produced in the rabbit, in the interior of the sac of collodion or of reed (the fine tubular membrane which lines the central cavity of the reed; segments of it may be used instead of collodion sacs). Is this particular microbe, then, the agent of the pleuro-pneumonic virus? Inoculation allows us to give a reply in the affirmative. In five Breton cows, inoculation of a small quantity of cultivations in sacs caused the development of an absolutely characteristic pleuro-pneumonic swelling. One of the cows succumbed with a formidable œdematous infiltration; the four others resisted. Two of these when reinoculated in a protected region with a strong dose (1 c.cm.) of pulmonary serum, showed absolutely no symptom, either local or general, while a fresh cow inoculated at the same time as these, for test purposes, with ten drops of the same serum, died twenty-two days after the inoculation. A third cow, when reinoculated four months after with 1 c.cm. of pulmonary serum from a very acute lesion, showed neither fever nor any local lesion. The fourth has not yet been reinoculated.

As we have said above, the cultivation extracted from a sac of collodion or of a reed, after being kept fifteen to twenty days in the peritoneum of a rabbit, however rich it may be, gives no culture when resown *in vitro* in the open air or in the vacuum, in any one whatsoever of the media, liquid or solid, generally used in bacteriology. One may, however, obtain cultures very similar to those of the sacs. But for that purpose we must use as our cultivation-liquid sterilized bouillon, not sown, which has been kept for some weeks in the interior of the sacs of collodion in the peritoneum of a cow or rabbit. This bouillon, though not sown, is itself modified by reason of the changes which take place through the coating-walls of the sacs; it becomes slightly albuminous; it acquires, above all, the faculty of being able to preserve for the culture *in vitro* some of the pleuro-pneumonic microbes. Only once did we obtain by sowing some drops of pleuro-pneumonic serum in freshly prepared peptonized bouillon a culture analogous to that of the sacs. At any rate, the bouillion sown after being kept seventy-two hours on the stove, showed the very slight opalescence and the small mobile and refringent specks which characterize this culture. But it was not possible for us to repeat the experiment, nor even to obtain a second culture after the one which chance had granted us.

Yet this observation confirmed us in the idea that the pleuro-pneumonic virus may be cultivated outside of the organism. We had, therefore, to find a favorable cultivation-medium. After long search we arrived at one. The liquid which gave us the best results was formed by the addition of a very small quantity of rabbit or cow serum to the peptone solution prepared by M. Louis Martin for the purpose of obtaining great quantities of diphtheritic toxin. The proportion of serum ought not to exceed one-twenty-fifth (about four drops for 5 c.c. of solution). No cultivation can be obtained if a solution of Witte or Chapotean peptone be employed; cultivation does not take place in presence of inert gases or in vacuum. The Martin serum bouillon does not only allow us to keep up the cultivation set on foot by the transference into sacs of collodion or of reed; it may also give a culture straight off when it is sown with a sprinkling of natural serum. The cultivation *in vitro* of the microbe of pleuro-pneumonia constitutes a great step in advance; one may now study its toxin and try to modify its virulence. It already has the advantage of preserving intact the pleuro-pneumonic virus, whilst it seemed to us to be perceptibly attenuated by the successive passages through the organism of the rabbit. But the degree of receptivity for the pleuro-pneumonic virus is so variable, even in individuals of the same race and age, that we do not venture to press the point. This question of the attenuation of the virus can only be settled by a great number of experiments. As to the first point—preservation of the virulence by means of the successive cultivations *in vitro*—it is clearly estab-lisced by the observation of one of the cows above mentioned. This cow, inoculated on February 26, 1898, with ten drops of a 6 per cent. culture, succumbed on March 19th with an enormous œdematous swelling in every way similar to those which inoculation of the most virulent pulmonary serum provokes.

The determination of the agent of the pleuro-pneumonic virulence has not only the interest of a difficulty surmounted; its range is much wider. It gives us the hope of succeeding likewise in the study of other viruses whose microbe has up till now remained unknown. What rendered the determination of the microbe of pleuro-pneumonia so difficult was its extreme tenuity on the one hand, and on the other hand, and especially, the limited conditions of its cultivation in an artificial medium. Now, we may be allowed to conceive of the existence of still smaller microbes, which, instead of remaining *within* limits of visibility, as is the case with this microbe, would be *without* these limits. In other words, it may be admitted that there exist microbes invisible to the eye of man. Well, even in the case of these microbes, their study remains possible, on the condition of there being found a medium which will be favorable to their cultivation. It will even be necessary in these attempts at culture not to attach too much importance to the modifications which take place in the appearance of transparency of the medium, in order to judge of failure or success. The cultivation of the microbe of pleuro-pneumonia is very fruitful; yet it does nothing but provoke a slight haze, a scarcely perceptible opalescence of the liquid; one is often obliged, in order to convince himself of the reality of the cultivation, to examine for comparison a tube of the same bouillon, not sown, placed beside it. The possibility may then be admitted of a microbian culture not modifying appreciably the appearance and limpidity of the medium. After that on the hypotheses that this same microbe would be one of those which are outside the limits of visibility, the only criterion of its presence and of its breeding by cultivation would be inoculation. Already certain experimenters may have found such cultivations; but they will not have recognized them, becsue the liquid having preserved its limpidity, they will deem it useless to inoculate with it. If such be the case the culture *in vivo* by means of collodion or reed-sacs, which has already rendered such services, does not seem to have said its last word; it is doubtless reserving new surprises for us.

CONCLUSIONS. The agent of the pleuro-pneumonic virulence consists of a microbe of extreme tenuity; its dimensions which are very inferior to those of the smallest known microbes, do not allow its form to be exactly determined after coloring. This microbe may be easily cultivated in sacs of collodion or reed inserted into the peritoneum of the rabbit. It does not give any cultivation when sown *in vitro* in the media ordinarily used. On the other hand, it can be easily cultivated when it is sown in Martin's bouillon-peptone, cow or rabbit serum being added in the proportion of one part of serum to twenty-five of bouillon.—*The Veterinary Journal and Annals of Comparative Pathology*, August, 1898.

